# CUTI-1: A Novel Tetraspan Protein Involved in *C. elegans* CUTicle Formation and Epithelial Integrity

**DOI:** 10.1371/journal.pone.0005117

**Published:** 2009-04-09

**Authors:** Julie-Anne Fritz, Carolyn A. Behm

**Affiliations:** Biochemistry & Molecular Biology, The School of Biology, College of Medicine, Biology and Environment, The Australian National University, Canberra, Australian Capital Territory, Australia; Centre for Genomic Regulation, Spain

## Abstract

The nematode cuticle is a tough extracellular matrix composed primarily of cross-linked collagens and non-collagenous cuticulins. It is required for nematode motility and protection from the external environment. Little is known about how the complex process of cuticle formation has been adapted to the specialized requirements of the nematode cuticle, which is structurally and compositionally unique from other organisms. The *C. elegans* gene *cuti-1* (CUTicle and epithelial Integrity) encodes a nematode-specific protein. We have shown that CUTI-1 is expressed in the epithelia and in seam cells. Within these tissues the expression of *cuti-1* mRNA cycles throughout development in line with the molting cycle, a process that involves synthesis of a new cuticle. In addition, knockdown of *cuti-1* by RNA interference (RNAi) results in worms that display post-embryonic phenotypes related to cuticle dysfunction and defects in epithelial integrity. This is one of the first reports of a nematode-specific protein involved in extracellular matrix formation. It provides further insight into how novel ways have evolved to regulate the formation of the cuticle, which is the primary protective barrier and skeletal component of nematodes.

## Introduction

The cuticle of the free-living nematode, *Caenorhabditis elegans*, is a tough extracellular matrix (ECM) composed primarily of cross-linked collagens and non-collagenous cuticulins [1]–[3]. It is important for providing body form to the worm and protecting it from the external environment. In addition, nematode locomotion requires force to be transmitted from the body wall muscles, through the hypodermis to the cuticle [4], [5].

The cuticle is synthesized and secreted by underlying epithelial cells, predominantly the hypodermis and seam cells, but also specialized epithelial cells that line the openings to the environment in the pharynx, vulva, anus and excretory pore. The cuticle is synthesized five times during development in a process that involves extensive tissue remodeling. The first cuticle is laid down during embryogenesis and re-synthesized underneath the old cuticle at each of the four larval molts. During this time, the seam cells, and to a lesser extent the hypodermal cells, accumulate large Golgi bodies and vesicles containing densely packed material. This is consistent with high levels of protein synthesis [6]. Simultaneously, the actin cytoskeleton reorganizes into circumferential bundles forming at the apical surface of the hypodermal cells [7]. These bundles then disappear during the final stages of cuticle formation. Consistent with the cyclical nature of cuticle formation and molting, the rate of synthesis of cuticular components and proteins involved in the assembly of the cuticle is high prior to molting and low between molts [8].

The process of cuticle formation and molting must be tightly regulated and controlled. Mutations in cuticle collagen genes, in enzymes involved in their processing, and in genes involved in the secretion of cuticle components result in a variety of cuticle defects. Worms with mutations in SEC-23, a component of the endoplasmic reticulum (ER) vesicle coat protein complex II (COPII) that is involved in ER to Golgi transport, display defects in cuticle secretion and lethality [9]: collagens remain within the hypodermis, and embryonic morphogenesis fails. Mutations in proteins involved in polarized secretion within the hypodermis also affect cuticle formation. Mutations in *che-14*, which encodes a probable component of the apical trafficking machinery in epithelial cells, result in an accumulation of vesicles close to the apical hypodermal membrane and a reduced cuticle thickness [10]. Unlike SEC-23, which is involved in the general secretory pathway and affects cuticle secretion in a gross manner leading to lethality, CHE-14 appears to be involved in the select secretion of apical membrane components since lethality is observed at a very low penetrance in *che-4* mutants [10]. Non-lethal mutations in other proteins, such as collagens, cause pleiotropic phenotypes that include defects in locomotion, for example the roller phenotype (helical twisting of the body) [11], gross morphological defects, such as the dumpy phenotype, and a dissociation of cuticular structures (the blistering phenotype) [1].

Here we report that silencing of expression of a novel tetraspan protein, CUTI-1, has a severe effect on the formation of the *C. elegans* cuticle. RNA interference (RNAi) targeted against *cuti-1* results in complex phenotypic defects including a dumpy body shape, impaired locomotion, abnormal cuticle morphology, and defects in seam cell function and fusion. In addition, we show that *cuti-1* is expressed exclusively in the epithelia where it is required during the period of cuticle formation. Evidence is also presented supporting an interaction of CUTI-1 with VPS-39, a protein thought to be involved in the tethering and fusion of vesicles.

## Materials and Methods

### Nematode strains and culture methods

*C. elegans* culture methods were as described [12]. The *C. elegans* strains Bristol N2 wild-type, JR1000 *ced-1(e1735)I*; *unc-119(ed4)III*; *wIs78* (*SCM::gfp*), *him-8(e1489)IV* and *daf-2(e1370)* were obtained from the *Caenorhabditis* Genetics Center (University of Minnesota, U.S.A.). The *vps-39* (T08G5.5) RNA interference clone was purchased from Geneservice Ltd. The strain TP12 (*col-19*::gfp) was a gift from Dr Antony Page (University of Glasgow, Scotland) and the strain FZ223 (*dlg-1*::gfp) was a gift from Dr Michel Labouesse (The Institute of Genetics and Molecular and Cellular Biology, France).

### Identification of CUTI-1 homologues

To identify potential CUTI-1 homologues, BLAST (Basic Local Alignment Search Tool) searches were conducted through NCBI (National Center for Biotechnology Information) and the EBI (European Bioinformatics Institute) using tBLASTx (translated query vs translated database). To identify *Brugia malayi* and *Haemonchus contortus* homologues, tBLASTx searches were conducted through TIGR (The Institute for Genomic Research; www.tigr.org) and The Wellcome Trust Sanger Institute (www.sanger.ac.uk) respectively.

### RNA interference (RNAi)

To construct the pCB76 RNAi feeding vector, primers were designed to PCR amplify the open-reading frame of *cuti-1* (ZC328.1). Primers used were 5′-ATGCCAAACGACCGGGTG-3′ and 5′-CTATCTGGTTTTGGATGACT-3′. The PCR product was sub-cloned into pGEM-Teasy (Promega) before a Not I restriction enzyme digest was carried out to clone the fragment into the RNAi feeding vector, pL4440 [13]. The identity of the fragment was confirmed by sequencing.

RNAi was carried out by bacterial feeding [14]. pCB19 was used as a non-specific dsRNA control as it has no homology to *C. elegans*. pCB19 contains a fragment of the *Arabidopsis thaliana* light harvesting complex gene (*Lhcb4.3*) cloned into the RNAi vector pL4440, and transformed into HT115 (DE3) *Escherichia coli* [15]. RNAi phenotypes were analysed after 48–72 hours at 21°C using a dissecting microscope and by mounting worms on 2% agarose pads which were viewed using a Leica light microscope fitted with Nomarski optics.

### Reporter gene analysis

Gateway® recombinatorial cloning was used to create a translational *cuti-1::gfp* construct [16]. Primers that contain the *att*B4 and *att*B1R recombination sites were designed to amplify approximately 2.5 kb upstream of the *cuti-1* initiation codon and the entire coding region except the stop codon. The ZC328 cosmid was used as template (kindly provided by The Wellcome Trust Sanger Institute). Primers used were 5′-CTCT-*att*B4-TGTTTCTTCATTGTACATAGT-3′ and 5′-attB1R-CTGGTTTTGGATGACTCTCT-3′. attB4 5′-GGGGACAACTTTGTATAGAAAAGTTG-3′ and attB1R 5′-GGGGACTGCTTTTTTGTACAAACTTGTC-3′. The fragment was amplified using AccuPrime Pfx polymerase (Invitrogen) in a standard reaction. The PCR product was cloned into pDONR(P4-P1R) and then pDEST-DD04 using BP and LR clonase (Invitrogen) respectively in one-third volume reactions. The identity and sequence of the fragment was confirmed by sequencing.

Microparticle bombardment of *C. elegans unc-119(ed3)* hermaphrodites was carried out to create the transgenic strain WT030 carrying an extrachromosomal array of *cuti-1::gfp* [17], [18]. Transgenic strains of *C. elegans* were mounted on 2% agarose pads and viewed using a Leica confocal microscope fitted with Nomarski optics.

### Semi-quantitative RT-PCR analysis

Semi-quantitative RT-PCR analysis was carried out to determine the temporal expression of *cuti-1* every two hours throughout development [8], [19]. Synchronous *C. elegans* first-stage larvae (L1s) were seeded onto standard NGM plates seeded with *E. coli* OP50 and incubated at 25°C. Approximately 8,000 L1s were seeded onto three plates for each time-point up to 28 hours, and 6,000 L1s onto two plates for the remaining time-points up to 40 hours. Worms were washed off plates every two hours using water, frozen in liquid nitrogen and total RNA extracted using TriZol reagent (Invitrogen) as per manufacturers instructions. For each time-point, 1 µg of total RNA was used as template in a first-strand cDNA synthesis reaction using the SuperScript™ First-Strand Synthesis System for RT-PCR (Invitrogen). 0.5–1.0 µl of cDNA was used as template in a multiplex PCR reaction to compare the expression of *cuti-1* with a control gene *ama-1*, which encodes RNA polymerase II [20], and *sqt-1*, which encodes a cuticle collagen [8]. Primers used were *cuti-1* 5′-ATGCCAAACGACCGGGTGGC-3′ 5′-TGAGTTCGACGTTCTGCTTG-3′, *ama-1* 5′-CAGTGGCTCATGTCGAGTTTCCAGA-3′ 5′-CGACCTTCTTTCCATCATTCATCG G-3′, and *sqt-1* 5′-TTCAACCAGCCAAAGACTCC-3′ 5′-CTTTCCTGGTCTTCCGTCTG-3′. Conditions used were 94°C for 2 minutes, followed by 25–30 cycles of 94°C 30 s, 55°C 30 s, 72°C 1 minute, and a final 72°C for 7 minutes. PCR products were detected by electrophoresis in 2% agarose gels, stained with ethidium bromide and visualized under UV light. Bands were quantified using ImageJ (Image Processing and Analysis Software in Java). The average value at a given time point was divided by the average *ama-1* value for that time point to generate relative values. Five biological replicates were analysed.

### Electron microscopy

*C. elegans* fed on pCB19 or pCB76 dsRNA-producing bacteria were prepared for scanning electron microscopy (SEM) [21]. Briefly, RNAi worms were washed off plates with PBS and fixed in 2.5% glutaraldehyde in PBS (pH 7.5) at 4°C for 2 hours. Worms were washed three times in PBS with a secondary fixation overnight in 1% osmium tetroxide at room temperature. Samples were washed five times in dH_2_O and dehydrated through an acetone series (30, 50, 75, 95, 100%). Washes were for 5 minutes each with the final three washes done in 100% acetone for 10 minutes each. Samples were critical point dried using CO_2_, mounted on stubs and coated with gold (20mA, 4 minutes). Samples were viewed using a Cambridge S360.

For transmission electron microscopy (TEM), RNAi worms were washed off plates with 0.1 M cacodylate buffer and fixed in 2.5% glutaraldehyde and 4% formaldehyde in buffer for 2 hours at room temperature. Worms were cut open immediately using a syringe. Worms were washed 3 times in cacodylate buffer for 10 min each wash, followed by a secondary fixation in 1% osmium tetroxide for 2 hours at room temperature. Three washes with dH_2_O were carried out before samples were dehydrated through an ethanol series (50, 70, 85, 95, 100%). Washes were for 15 min each with the final three washes done in 100% ethanol for 15 min each. Samples were infiltrated as follows: two changes of 100% acetone for 15 min each; 2∶1 change of acetone : epon araldite for 2–3 hr; 1∶1 change of acetone : epon araldite overnight at 4°C; 1∶2 change of acetone : epon araldite for 2–3 hr; three changes in pure epon araldite for 3–4 hr each. Samples were embedded in blocks and baked overnight at 60°C. Blocks were trimmed using a glass knife and 70 nm sections cut using a diamond knife. Sections were mounted on copper grids and stained with 6% uranyl acetate for 20 min, washed thoroughly with dH_2_O and stained with lead citrate for 15 min. Samples were washed thoroughly with dH_2_O, dried, and viewed using a Hitachi H7100FA transmission electron microscope. Five individuals were viewed for each sample.

### Hoechst staining of nuclei

Hoechst staining was carried out as described [22]. Briefly, RNAi worms were washed off plates with M9 buffer and incubated in M9 buffer supplemented with 1 µg/ml Hoechst 33258 (Sigma) at room temperature for 15 min with gentle agitation. Worms were washed three times with M9 buffer to remove dye. Staining of nuclei was visualized using fluorescence microscopy with a Leica microscope fitted with Nomarski optics and a FITC filter. All images were acquired using the same settings and exposure.

### Yeast two-hybrid analysis

For generation of DB-X clones, Gateway® recombinatorial cloning was used [23]. Primers that contain the *att*B1.1 and *att*B2.1 recombination sites were designed to amplify the *cuti-1* open-reading frame (ORF) in addition to regions outside of predicted trans-membrane regions to eliminate these from the analysis. Primers used to amplify the ORF were 5′-*att*B1.1-GCCAAACGACCGGGTGGCTCCA-3′ and 5′-*att*B2.1-TCTGGTTTTGGATGACTCTCT-3′. Primers used to amplify the first, second, third, fourth and fifth cytoplasmic regions (CUTI-I, II, III, IV, V) were as follows:

**CUTI-I**: 5′-*att*B1.1-GCCAAACGACCGGGTGGCTCCA-3′ 5′-*att*B2.1-TGTATAATGCATTGAATTACA-3′;
**CUTI-II**: 5′-*att*B1.1-GATGAGCCACAAATCGGGA-3′ 5′-*att*B2.1-CCATTTTCCAATTGAATC;
**CUTI-III**: 5′-*att*B1.1-GTGGAAAGAAAATCCACAGATG-3′ 5′-*att*B2.1-AAATTTCAATTTTGATCCTAG-3′;
**CUTI-IV**: 5′-*att*B1.1-GCAATTCACAAGACAAGTCTTT-3′ 5′-*att*B2.1-TGGTCCGTAATCTTGTTCAAT-3′;
**CUTI-V**: 5′-*att*B1.1-GATTCAAGGAGCTGCTGATTAT-3′ 5′-*att*B2.1-TCTGGTTTTGGATGACTCTCT-3′.

The *att*B sequences used were as follows: *att*B1.1: 5′-GGGGACAACTTTGTACAAAAAGTTGGCAT-3′ and *att*B2.1: 5′-GGGGACAACTTTGTACAAGAAAGTTGGGTA-3′. CUTI-II was not successfully amplified. BP and LR recombination reactions were carried out using pDONR223 and pDEST-DB, respectively, to generate DB-X fusions. The identity and sequence of fragments was confirmed by sequencing.

Individual DB-X plasmids containing either the *cuti-1* ORF or fragments were transformed into the yeast strain MaV203 [24]. Yeast two-hybrid screening was carried out by transforming each DB-X-containing MaV203 strain with 20 µg of a *C. elegans* AD-Y cDNA library (AD-wrmcDNA) kindly provided by Dr Jean-François Rual [24]. Cotransformants were assayed for growth on selective media either lacking uracil (-URA) or histidine (-HIS), in addition to assaying the production of β-galactosidase (X-GAL). Potential positives were picked and only those that passed all three phenotypic tests were stored as glycerol stocks and sequenced.

### GST-pull down experiments

GST fusion proteins for pull-down experiments were expressed in *E. coli* strain BL21 (DE3) (Novagen). The *att*B1.1 and *att*B2.1 sites, described above, were replaced with *BamH* I and *Not* I restriction sites to amplify the CUTI-1 cytoplasmic regions. These products were cloned into the pGEX-4T-3 expression vector (Amersham Biosciences) using standard recombinant techniques and confirmed by sequence analysis. Cloning of the CUTI-III region was unsuccessful.

To express GST fusion proteins, one liter cultures of BL21 (DE3) containing pGST::CUTI-II, pGST::CUTI-IV, or GST alone, were grown in LB supplemented with ampicillin, shaking at 37°C, until an OD_600_ of 0.6 was reached. Expression of proteins was induced with 0.1 mM IPTG at 30°C for a further two hours with shaking. Cells were harvested by centrifugation at 7,700×*g* for 10 min at 4°C, then resuspended in 50 ml of ice-cold PBS and lysed through two passes in a French Press at 20,000 psi. Lysed cells were rocked for 30 min at 4°C in 1% Triton X-100. Cellular debris was pelleted by centrifugation at 12,000×*g* at 4°C for 10 min and the supernatant, containing soluble GST fusion proteins, was frozen at −80°C until use. GST fusion proteins were purified using Glutathione Sepharose 4B according to the GST Gene Fusion System Handbook (Amersham Biosciences).

The VPS-39 ‘prey’ protein used for pull-down experiments was synthesized by *in vitro* transcription and translation using the TNT® Coupled Reticulocyte Lysate System (Promega). An in-frame 3′ end of VPS-39, which includes the clathrin heavy chain repeat (CLH) motif, was amplified using primers with *Xba* I and *Not* I restriction sites on the 5′ and 3′ ends respectively. In addition, the 3′ primer contained a FLAG epitope to enable detection of the *in vitro* translated protein with anti-FLAG antibodies. Primers used to amplify VPS-39 were 5′-CTCTCTCGAGATGGCTAGAAATTTAAATCG-3′ and 5′-CTCTGCGGCCGCTCACTTGTCGTCATCGTCTTTGTAGTCATTTCTGTTTCCTCCTTGAG-3′.

Pull-down experiments were performed by incubating *in vitro* translated proteins with GST, GST::CUTI-II or GST::CUTI-IV protein immobilized onto Glutathione Sepharose 4B media for 4 hr at 4°C. The Sepharose was washed four times with PBS to remove unbound protein. GST-‘prey’ protein complexes were then eluted from the Glutathione Sepharose by the addition of 10 mM reduced glutathione. After a 10 min incubation at room temperature, GST-‘prey’ protein complexes were recovered by centrifugation of the sample at 500×*g* for 5 min and collection of the supernatant. The supernatant was heated at 70°C in 15 µl SDS-PAGE loading buffer for 10 min before samples were run in NuPAGE Bis-Tris SDS-PAGE gels (Invitrogen) and transferred to an iBlot membrane (Invitrogen) for western blot analysis. *In vitro* translated proteins were detected using mouse M2 anti-FLAG antibodies (Sigma) diluted 1,000∶1. Primary antibodies were detected using anti-mouse secondary antibody conjugated to peroxidase (Sigma) diluted 10,000∶1. Membranes were incubated with Chemiluminescent Peroxidase Substrate-3 (Sigma) and exposed to Hyperfilm (Amersham Biosciences) to detect peroxidase activity.

## Results

### *cuti-1* encodes a novel tetraspan protein

**Figure 1:**
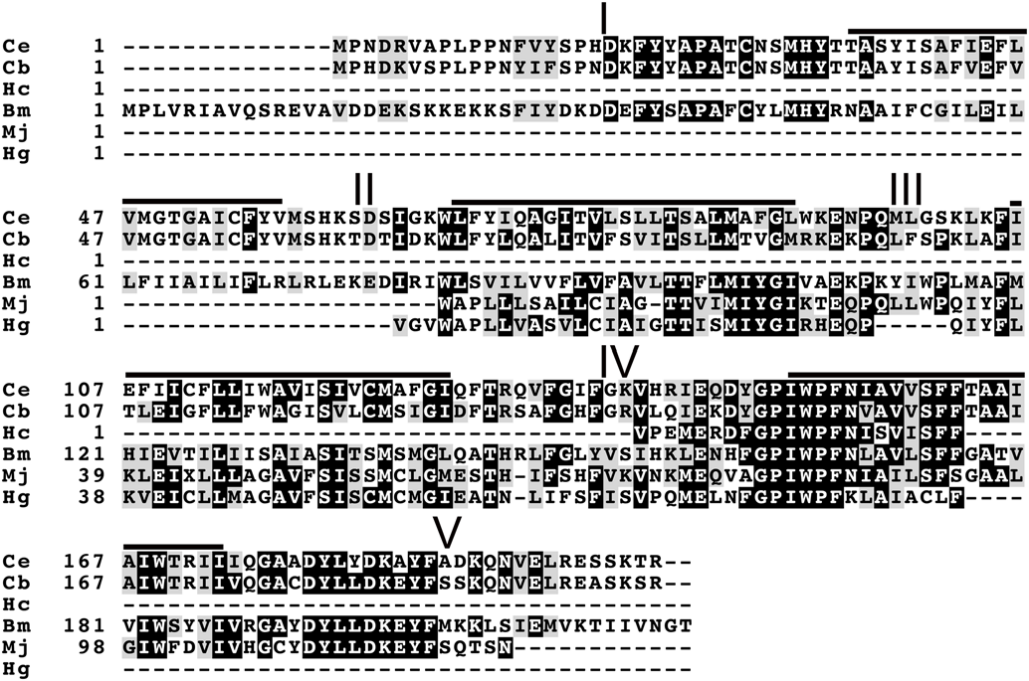
Multiple sequence alignment of CUTI-1 with known nematode homologues. A translated BLAST analysis was carried out through the European Bioinformatics Institute server. This identified homologues in *C. briggsae* (Cb; CBG12615), *Meloidogyne javanica* (Mj; BE578004), and *Heterodera glycines* (Hg; CB824616). In addition, a translated BLAST analysis was carried out against the *Haemonchus contortus* genome through the Wellcome Trust Sanger Institute and the *Brugia malayi* genome through The Institute for Genomic Research. This identified the partial *H. contortus* (Hc) homologue (haem-257m03.q1k) and the *B. malayi* homologue (Bm; Bm1_49050). Solid bars denote predicted transmembrane domains of *C. elegans* (Ce) CUTI-1. Roman numerals denote cytoplasmic regions I–V.

*cuti-1* (ZC328.1) is predicted to encode a four pass transmembrane protein of 202 amino acids in length with no signal peptide. This protein has no regions of homology to known protein families, motifs or functional sites as predicted by InterProScan. Using a cut off expect value of 10^−5^, tBLASTx homology searches revealed that this protein has no significant similarity to proteins from organisms outside of the Nematoda. At the amino acid level, CUTI-1 shares 72% identity with its closest homologue from *C. briggsae*, 37% with a protein from the Clade II filarial parasite, *Brugia malayi*, 34% and 44% with proteins from the Clade IV plant parasitic nematodes *Heterodera glycines* and *Meloidogyne javanica* respectively, and 68% with a protein from the Clade V veterinary parasite, *Haemonchus contortus* (Figure 1).

### *cuti-1(RNAi)* worms display defects in cuticle formation and epithelial integrity

**Figure 2:**
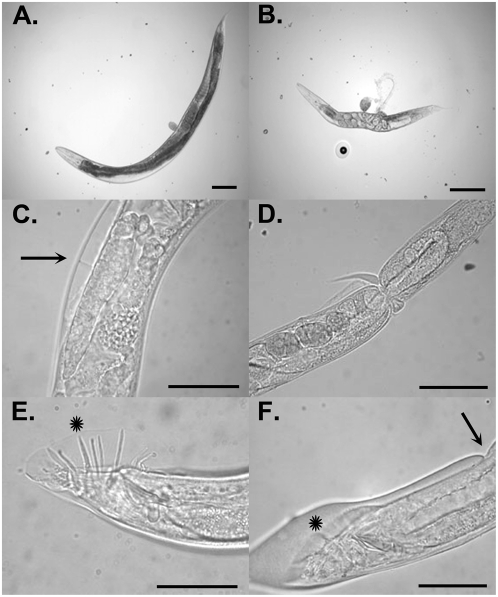
Postembryonic RNAi phenotypes observed following knockdown of expression of *cuti-1* in *C. elegans*. Nomarski micrographs of *C. elegans* fed on dsRNA-expressing bacteria for 3 days at 21°C. A. A gravid N2 hermaphrodite fed on control dsRNA. B–D. N2 *C. elegans* fed on *cuti-1* dsRNA displaying a variety of defects related to cuticle dysfunction that includes: dumpy body morphology and ruptured vulva (B), cuticle blistering (C) and cuticle constrictions caused by the previous cuticle wrapping around the body (D). E. A *control(RNAi)*;*him-8(e1489)* male showing fully elongated sensory rays (asterisk). F. A *cuti-1(RNAi)*;*him-8(e1489)* male showing sensory rays that have not fully elongated (asterisk) and blistering of the cuticle (arrow). Scale bars 50 µm.

When wild-type *C. elegans* were fed on bacteria producing *cuti-1* dsRNA, all worms were dumpy (dpy) and un-coordinated (unc) compared with controls (Figure 2a-b). Worms were able to move in a sinusoidal manner but could not progress forward or backward (the ‘skiddy’ phenotype). The cuticle of *cuti-1(RNAi)* worms also became blistered, where the outer layers of cuticle dissociated from the body (Figure 2c). Approximately 10% of worms displayed excessive tapering of the cuticle in the posterior region of the body in addition to retaining old cuticle wrapped around various regions of the body forming tight constrictions (Figure 2d). These constrictions presumably occur due to the inability of the worm to effectively free itself from the old cuticle during the molting process. Males also displayed abnormalities in formation of the sensory rays, where the rays did not fully extend and the cuticular fan was reduced in size compared with controls (Figure 2e–f).

**Figure 3:**
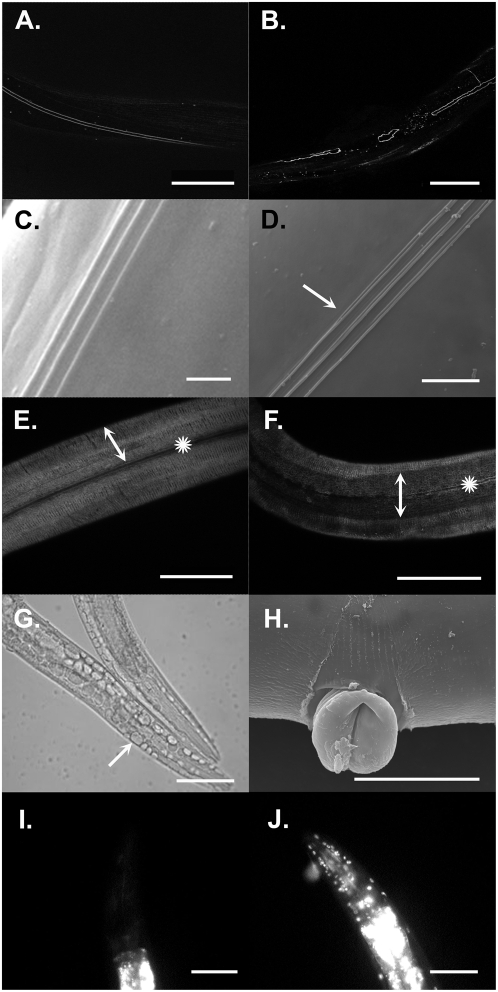
*cuti-1(RNAi)* worms display defects in seam cell function and epithelial integrity. A, B. Confocal micrographs of the seam cell boundaries of adult *control(RNAi)*;*dlg-1::gfp* and *cuti-1(RNAi)*;*dlg-1::gfp* worms respectively. *cuti-1(RNAi)*;*dlg-1::gfp* worms either failed to form a seam cell syncytium or failed to maintain the cell-cell contact between seam cells (B). C, D. Scanning electron micrographs of N2 worms fed control dsRNA and *cuti-1* dsRNA respectively. The lateral alae secreted by the underlying seam cells in *cuti-1(RNAi)* worms were bifurcated (arrow) (D). E, F. Confocal micrographs of adult *control(RNAi)*;*col-19::gfp* and *cuti-1(RNAi)*;*col-19::gfp* worms respectively. COL-19 is an adult-specific collagen that is expressed in the lateral alae (asterisks) and circumferential annuli (double-headed arrows) of the cuticle (E). Using COL-19 as a marker, the alae (asterisks) and annuli (double-headed arrow) appear highly disorganized in *cuti-1(RNAi)*;*col-19::gfp* adults (F). G. Nomarski micrograph of a *cuti-1(RNAi)* L4 worm showing vacuolation of the hypodermis (arrow). H. Scanning electron micrograph of an adult *cuti-1(RNAi)* worm showing a protruding vulva. I, J. Fluorescent micrographs of the anterior region of N2 adults fed control dsRNA and *cuti-1* dsRNA respectively. Worms were soaked in a 1 µg/ml solution of Hoechst 33258 (Sigma) for 15 mins. Hoechst 33258 is a membrane impermeable dye that fluoresces when bound to DNA. *Control(RNAi)* worms do not show fluorescence of nuclei when soaked in a solution of Hoechst 33258 (I). In contrast, nuclei of *cuti-1(RNAi)* worms fluoresce because of reduced barrier function of the cuticle and hypodermis (J). Scale bars 50 µm, except in C and D where scale bars 10 µm.

The sensory rays are involved in locating the hermaphrodite vulva during copulation and are secreted by the lateral hypodermal cells, the seam cells. The seam cells are also responsible for secretion of the lateral cuticle and specialized cuticular structures called alae, which are tri-laminate structures present only on the surface of first-stage larvae, adults and dauer larvae, a long-lived alternative life stage of *C. elegans*. Seam cells undergo several divisions during development that are coupled to each larval molt. At the beginning of each larval stage, the posterior daughter cell of each seam cell division becomes a seam cell and re-forms cell-cell contact with its neighboring cell, while the anterior daughter cell becomes detached and fuses with the hypodermal syncytium, hyp 7 [25]. During the molt to adulthood, the seam cells fuse completely to form a syncytium [25]. To investigate seam cell function, the morphology and number of seam cells was examined using two transgenic strains of *C. elegans*, *SCM::gfp* (JR1000) and *dlg-1::gfp* (FZ223), which express GFP in the nuclei and at the apical surface of seam cells respectively [26], [27]. Both strains were fed on bacteria induced to express *cuti-1* dsRNA. Using the SCM::GFP marker, no abnormalities were seen with the division or number of seam cells specified throughout development (results not shown). Examination of DLG-1::GFP, however, revealed abnormalities with seam cell fusion and morphology in the adult (Figure 3a–b). The seam syncytium did not maintain its linear appearance and failed to fuse in some instances, suggesting that the cells were unable to maintain or form correct cell-cell contacts.

In order to investigate defects in seam cell function further, the alae secreted by the seam cells were observed in the adult stages using scanning electron microscopy. The alae of *cuti-1(RNAi)* worms were found to be either absent (not shown) or to bifurcate in some instances, further demonstrating abnormal seam cell function (Figure 3c–d). In addition, a *col-19::gfp* transgenic strain of *C. elegans* was used to investigate other defects in the cuticle surface. COL-19 is an adult-specific collagen expressed in the lateral alae and circumferential annuli of the cuticle; it has been used as a marker for changes in cuticle modification and assembly [21]. Expression of GFP was weak and disorganized in *cuti-1(RNAi)*;*col-19::gfp* worms compared with controls (Figure 3e–f). Expression in the alae was present but discontinuous in regions, perhaps due to bifurcation of the alae. Expression was also observed in the annuli of the lateral hypodermis but was not linear in appearance compared with the organized patterning of the annuli seen in controls.

In addition to defects in seam cell function and fusion, *cuti-1(RNAi)* worms displayed further deficiencies in maintaining epithelial integrity. Vacuolation of the hypodermis was observed and as *cuti-1(RNAi)* hermaphrodites reached gravidity, the vulva began to protrude and eventually ruptured, leading to death (Figure 3g–h). This could be due to loss of vulval-hypodermal cell contact or excessive stress being placed on the already weak vulval epithelia during egg-laying. The degeneration of the hypodermis was confirmed using the membrane-impermeable dye Hoechst 33258, which fluoresces when bound to DNA and thus stains nuclei of permeable, but not impermeable, living cells. When *cuti-1(RNAi)* worms were suspended in buffer containing Hoechst 33258, all worms showed fluorescence of nuclei compared with none of controls (Figure 3i–j). This confirms that silencing of *cuti-1* expression causes the hypodermal membrane and cuticle to become highly permeable and no longer able to provide a protective barrier from the environment.

### Ultra-structural analysis reveals defects in formation of cuticle layers and vesicle transport

**Figure 4:**
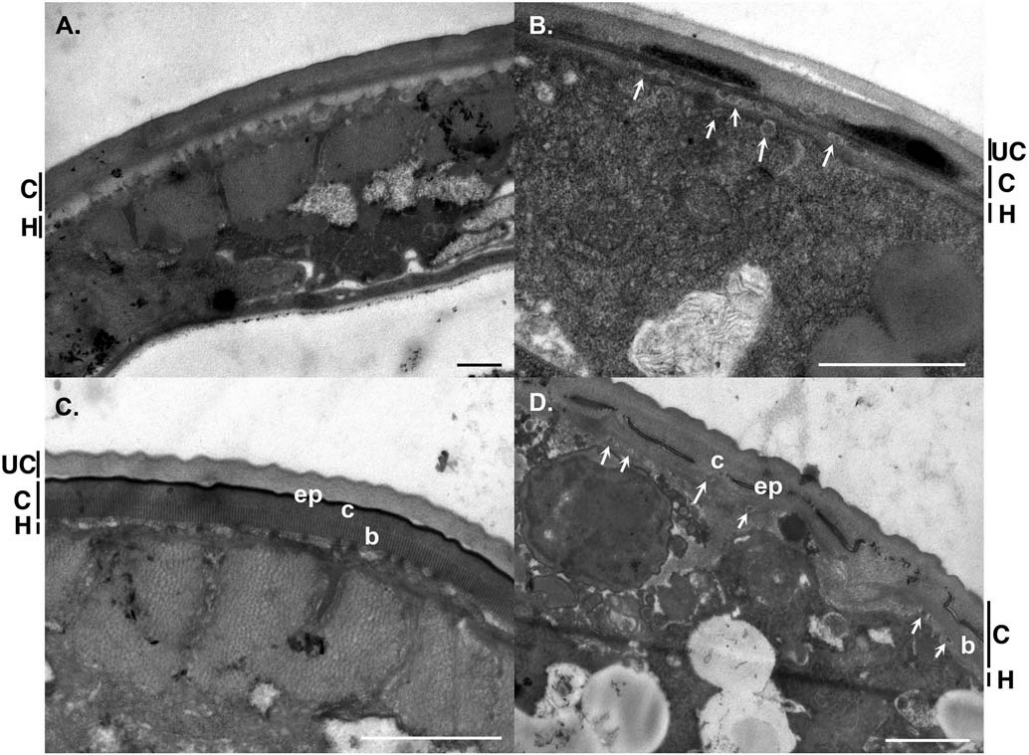
Ultrastructural analysis of *cuti-1(RNAi)* adults and dauer larvae. Transmission electron micrographs of N2 (A, B) and *daf-2(e1370)* worms (C, D). A, B. Adult N2 *C. elegans* fed control and *cuti-1* dsRNA respectively. In *cuti-1(RNAi)* worms electron dense material accumulated within the cuticle and vesicles or vesicle-like structures accumulated within the hypodermis (arrows) (B). *cuti-1(RNAi)* worms also tended to retain cuticle from the previous developmental stage. C, D. *daf-2(e1370)* dauers fed control and *cuti-1* dsRNA respectively. In *cuti-1(RNAi)* dauers, the epicuticle (ep) was present within the cortical layer (c) instead of at the apical surface. In addition, the cortical layer was expanded and had a granular appearance. The basal layer (b) was present, but was not striated compared with *control(RNAi)* worms. Vesicles or vesicle-like structures were also evident within the hypodermis (arrows). C, cuticle; H, hypodermis; UC, unshed cuticle. Scale bars 1 µm.

Analysis of the ultra-structure of *cuti-1(RNAi)* worms was undertaken to further investigate the defects in cuticle formation. The cuticle formed by adult *cuti-1(RNAi)* worms showed an accumulation of electron dense material within the layers. In addition, these worms did not have lateral alae, consistent with previous observations using scanning electron microscopy. Vesicles or vesicle-like structures accumulated within the hypodermis, suggestive of a defect in vesicular transport. Unshed cuticle was also present in some worms, in addition to a thickening of the hypodermal layer (Figure 4a and b). To determine if similar structural defects would be observed in the cuticle of other developmental stages, the ultra-structure of the cuticle formed in dauer larvae was examined. The dauer is a long-lived, alternative developmental stage of *C. elegans*. Dauer larvae also possess a cuticle that is structurally distinct from other life-stages, with a thick, highly striated basal zone and specialized lateral alae. Dauers for this analysis were generated by feeding *daf-2(e1370)* mutants on *cuti-1* dsRNA and incubating the worms at the restrictive temperature of 25°C. Similar to wild-type worms fed on *cuti-1* dsRNA, 98% of *cuti-1(RNAi)*;*daf-2(e1370)* worms were dumpy compared with controls (not shown). Ultra-structural analysis revealed gross defects in the cuticles of these worms. The striated, basal layer of the cuticle was present, but had collapsed in some regions with the striations no longer being defined. The cortical layer was wider and had a granular appearance compared with *control(RNAi)*;*daf-2(e1370)* worms (Figure 4c and d). In some regions, the cortical layer showed accumulation of electron dense material and the placement of the epicuticle was mis-specified as it was within the cortical layer instead of at the apical surface of the cuticle. Further, *cuti-1(RNAi)*;*daf-2(e1370)* worms did not have any alae compared with *control(RNAi)*;*daf-2(e1370)* worms, consistent with the defects in alae production observed in adults.

### CUTI-1 is expressed in the epithelia and its expression cycles during postembryonic development

**Figure 5:**
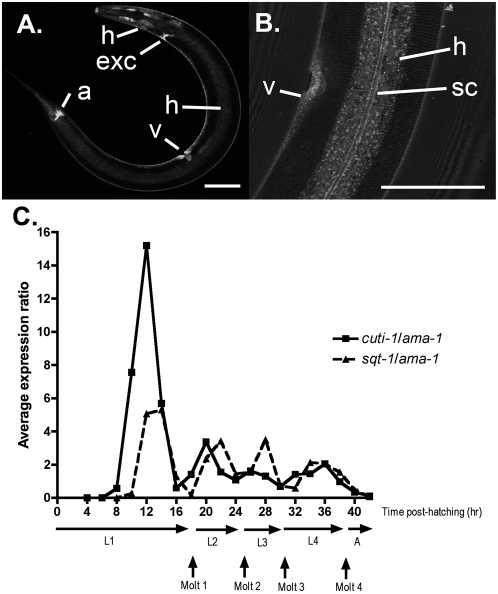
The spatial and temporal expression of CUTI-1::GFP and *cuti-1* mRNA in *C. elegans*. A, B. Confocal micrographs of the expression pattern observed in CUTI-1::GFP transgenic *C. elegans*. A. A transgenic L4 worm. GFP expression is observed in the hypodermal (h) cells of the head, vulva (v) and the main body syncytia, hyp 7, which also surrounds the opening of the excretory canal (exc) and the anus (a). B. In addition, GFP is present in the seam cells and at the seam cell boundary (sc). The transcription of *cuti-1* cycles throughout development, peaking prior to each molt (C). This expression pattern is similar to that of the collagen, *sqt-1*, which is up-regulated two hours prior to each larval molt [36]. Scale bars 50 µm.

The spatial expression of CUTI-1 was examined using a translational GFP fusion. Transgenic *C. elegans* expressing the *cuti-1::gfp* translational transgene (WT030) displayed GFP expression in the developing hypodermis of mid- and late-stage embryos (not shown). GFP expression was also observed in the epithelia throughout development until the fourth-stage larva. At no time was GFP observed in adult stages. Expression was seen exclusively in the cytoplasm of the hypodermis, in the vulval and anal epithelium, and in the excretory pore (Figure 5a). Within the hypodermis, GFP expression was observed at small discrete points, possibly corresponding to vesicle-like structures (Figure 5b). Strong expression was also observed in the seam cells and at the seam cell boundary (Figure 5b). All of these tissues are covered by cuticle or involved in cuticle synthesis and secretion [2], [3], [28].

To investigate the temporal expression of *cuti-1*, semi-quantitative RT-PCR was performed on total RNA extracted from wild-type *C. elegans* every two hours during development from four hours post-hatch until adulthood. As control transcripts, the *C. elegans* genes *ama-1* and *sqt-1* were used. *ama-1* encodes RNA polymerase II [20]. It has been shown previously that expression levels of *ama-1* remain relatively constant throughout development, and it is therefore an appropriate control gene for comparing expression levels of a target gene during different stages of the *C. elegans* life-cycle [8], [19], [29]. *sqt-1* encodes a cuticle collagen that is up-regulated about two hours before each larval molt [8]. This gene serves as an internal control for the approximate time of each molt. The expression of *cuti-1* was found to rise and fall throughout development, broadly peaking at 12, 20, 26 and 36 hours post-hatch, prior to each larval molt (Figure 5c). This expression pattern was similar to that of *sqt-1* mRNA, which peaked about two hours after each *cuti-1* peak throughout development, except at the L4 to adult molt where both mRNAs appear to peak simultaneously.

### CUTI-1 interacts with VPS-39, a homologue of the yeast vacuolar assembly/sorting protein

**Figure 6:**
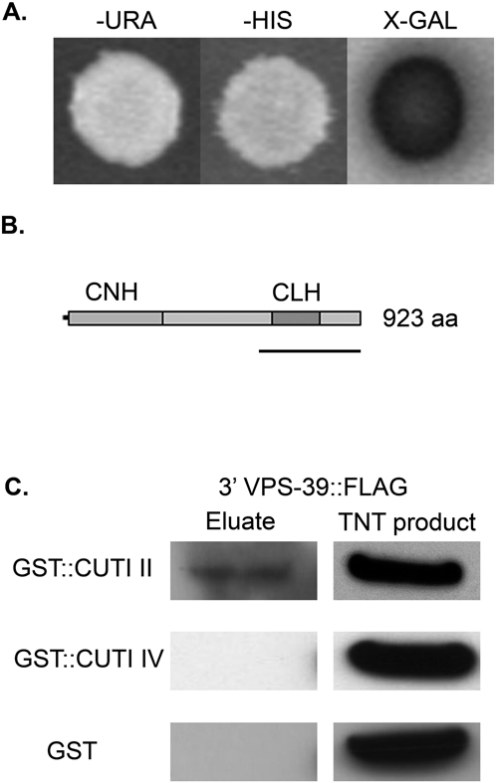
CUTI-1 interacts with the vesicle protein, VPS-39. A. Full-length CUTI-1 and each of the cytoplasmic regions, excluding region II, were fused to the Gal4 DNA binding domain (DB) and used to probe a cDNA library of proteins fused to the Gal4 activation domain (AD). Cotransformants were assayed for growth on selective media either lacking uracil (-URA) or histidine (-HIS), in addition to assaying the production of β-galactosidase (X-GAL). (A) shows an example of a colony containing CUTI-I and VPS-39 that tested positive for an interaction under all phenotypic conditions used. B. Schematic representation of *C. elegans* VPS-39 showing domain architecture. *C. elegans* VPS-39 is a 923 amino acid (aa) protein that contains an amino-terminal citron homology domain (CNH) and a carboxyl-terminal clathrin heavy chain repeat (CLH) motif. Underline denotes the region fused to AD in yeast two-hybrid experiments. C. The II and IV cytoplasmic regions of CUTI-1 were fused to GST and immobilised onto glutathione beads. The carboxyl terminus of VPS-39 fused to a FLAG epitope (VPS-39::FLAG) was then incubated with the GST protein for three hours to allow interactions to occur. Unbound VPS-39::FLAG was then removed before any bound interacting proteins were removed and detected by carrying out an anti-FLAG immunoblot. As a control, GST was used to determine if GST alone could interact with VPS-39::FLAG. TNT, 3′ VPS-39::FLAG product synthesized *in vitro* using the TNT® Coupled Reticulocyte Lysate System (Promega).

To identify proteins that potentially interact with CUTI-1, yeast two-hybrid experiments were conducted. Because nuclear localization is necessary to obtain reporter gene expression in this method, proteins with hydrophobic membrane domains, such as CUTI-1, may not be amenable to this localization. In order to circumvent this problem, we used both the full-length protein and fragments of the CUTI-1 coding sequence that excluded predicted transmembrane domains to screen a mixed-stage *C. elegans* cDNA library. Using this technique, all CUTI-1 fragments were found to interact with VPS-39 (Vacuolar Protein Sorting factor 39). These interactions were confirmed using a pair-wise yeast two-hybrid approach (Figure 6a). The *vps-39* cDNA fragment shown to interact with CUTI-1 was found to encode a region in the C terminus of the predicted protein that encodes a clathrin heavy chain repeat (CLH) motif (Figure 6b). GST pull-down experiments were used to confirm the putative interaction between fragments of CUTI-1 and the CLH motif of VPS-39. Recombinant GST::CUTI-1 fragment II expressed in *E. coli* strain BL21 (DE3) was able to bind the CLH motif of VPS-39, which was synthesized *in vitro* (Figure 6c). Together, these experiments provide evidence that CUTI-1 may interact physically with VPS-39. RNAi was carried out on *vps-39* in order to determine if a knockdown in this gene would result in similar cuticular defects as seen in *cuti-1(RNAi)* worms. *vps-39(RNAi)* worms did not, however, display phenotypes similar to those observed in *cuti-1(RNAi)* worms. Instead, all *vps-39(RNAi)* worms resembled *control(RNAi)* worms.

## Discussion

*cuti-1* encodes a novel tetraspan protein that does not appear to have homologues outside the Phylum Nematoda. We have demonstrated that CUTI-1 plays an important role in formation of the nematode cuticle. A reduction in expression of *cuti-1* by RNA interference results in complex phenotypic defects including a dumpy body shape, the inability to locomote, abnormal cuticle morphology and permeability, and defects in seam cell fusion and function. Consistent with this, CUTI-1 is present in the hypodermis and seam cells, the main sites of cuticle synthesis. The expression of *cuti-1* mRNA also cycles during development, consistent with a protein involved in cuticle formation.

Furthermore, ultrastructural analysis of *cuti-1(RNAi)* worms showed an accumulation of dense material within the cuticle, suggesting that cuticle components had not been secreted correctly. In addition, vesicle-like structures accumulated within the hypodermis, suggesting a defect in transport mechanisms that are important for correct formation of the cuticle. A similar accumulation of vesicles within the hypodermis is also seen in *che-14* mutants [10]. CHE-14 is hypothesized to be essential in the apical secretory pathway of some epithelial cells for exocytosis of proteins with lipid modifications. We suggest that CUTI-1 may function in a similar or related pathway for apical secretion, because the RNAi phenotype is different from those observed for proteins that function in generalized cuticle secretory processes, such as SEC-23. *sec-23(RNAi)* worms lack the ability to transport proteins from the ER to the Golgi, and hence have a defect in global secretion. They display a fragile cuticle that often ruptures upon handling, the cuticle surface is disorganized, and collagens such as DPY-7 are retained within the hypodermis instead of being secreted to the cuticle. Embryonic lethality in a *sec-23* mutant is also observed, due to an inability to secrete a functional first cuticle [9]. In contrast, *cuti-1(RNAi)* worms do not rupture upon handling or when suspended in water or buffer (data not shown). In addition, collagens such as COL-19 continue to be secreted to the cuticle, although this collagen displays aberrant localization. Similar to a *sec-23* mutant, however, we have observed in preliminary experiments that embryos carrying a deletion in *cuti-1* (*tm1388*) frequently arrest prior to gastrulation during embryogenesis (data not shown). Given that RNAi often does not result in a complete loss-of-function phenotype, the role of CUTI-1 during embryogenesis needs to be investigated in greater detail in *tm1388* mutants to fully discount a role for CUTI-1 in the general secretion of cuticle components.

We have provided evidence of an interaction of CUTI-1 with VPS-39. CUTI-1 may be involved in the docking or fusion of vesicles either within the epithelia or to the epithelial membrane. VPS-39 derives its name from its homology to the human protein VAM-6 and the yeast protein VPS-39. Both the human and yeast proteins have been shown to play important roles in vesicle tethering and fusion [30], [31]. Absence of CUTI-1 may lead to an unstable association of vesicles, which would result in cuticle components not being incorporated into the new cuticle in a uniform manner. This is consistent with the variety of RNAi defects related to cuticle dysfunction and the accumulation of vesicle-like structures seen in *cuti-1(RNAi)* worms. In addition, it is possible that CUTI-1 carries out these functions independent of VPS-39, as suggested by their different RNAi phenotypes. In our hands, *vps-39(RNAi)* worms do not display phenotypes related to defects in cuticle formation. Further, analysis of the *vps-39* deletion mutant (*tm2253*) by Lackner and colleagues revealed defects in germline apoptosis, but not cuticle formation [32]. CUTI-1 may be involved in transport of certain cuticle components independent of vesicles, such as by associating with lipid rafts, similar to the tetraspan protein VIP17/MAL in mammalian epithelial cells [33] or CHE-14 [10]. Involvement of lipid rafts in cuticle secretion in *C. elegans* has not yet been shown and remains speculative.

The main deficiency in *cuti-1(RNAi)* worms may be in the epithelia rather than in the process of cuticle component secretion. Little is known about the role of the epithelia during cuticle formation. Studies have shown that during the time of cuticle secretion, circumferentially oriented actin bundles are transiently connected to the cuticle during each postembryonic molt [7]. During embryogenesis, these bundles are thought to act as a template for the first cuticle layer. A disruption of this process, or instability of the epithelial membrane during this time, could lead to abnormal patterning of the subsequent cuticle. A role for CUTI-1 in maintaining the stability of the hypodermis seems unlikely, however, as worms with mutations in MUP-4 and MUA-3, proteins known to be essential for the attachment of the hypodermal surface to the cuticle, do not show the cuticle defects we observed in *cuti-1(RNAi)* worms [34], [35]. Instead, absence of MUP-4 and MUA-3 results in failed embryonic morphogenesis and post-embryonic paralysis due to the loss of mechanical coupling between the body wall muscles, through the hypodermis, to the cuticle.

*cuti-1* encodes an apparently nematode-specific protein. Potential orthologues have been identified in *C. briggsae* and a number of parasitic species across the Phylum. Proteins such as CUTI-1 and its orthologues may have evolved to adapt the complex process of cuticle formation to the specialized requirements of the nematode cuticle, which is structurally and compositionally unique from other organisms [3]. The process of cuticle formation is essential to the development and viability of all nematodes. The evolution of novel ways to regulate the formation of the cuticle, which is the primary protective barrier and skeletal component, may provide nematodes with greater flexibility when dealing with environmental change and host immune attack.

It is likely that many more nematode-specific proteins are involved in the process of cuticle formation. Identification of these proteins will provide further insight into how formation of a complex extracellular matrix, such as the nematode cuticle, has been modified to suit the requirements of the organism. In addition, some of the proteins may provide useful drug targets in the control of parasitic nematodes of medical and economic importance.
